# Tuning the Density of Zwitterionic Polymer Brushes on PET Fabrics by Aminolysis: Effect on Antifouling Performances

**DOI:** 10.3390/polym12010006

**Published:** 2019-12-18

**Authors:** Emanuela Lorusso, Wael Ali, Michael Leniart, Beate Gebert, Markus Oberthür, Jochen S. Gutmann

**Affiliations:** 1 Deutsches Textilforschungszentrum Nord-West ÖP GmbH, 47798 Krefeld, Germany; jochen.gutmann@uni-due.de; 2 Department of Physical Chemistry and Center of Nanointegration (CENIDE), University Duisburg-Essen, 45141 Essen, Germany; ali@dtnw.de; 3 Deutsches Textilforschungszentrum Nord-West gGmbH, 47798 Krefeld, Germany; michaelleniart@gmail.com (M.L.); beate.gebert@dtnw.de (B.G.); 4 Department of Design, Hochschule für Angewandte Wissenschaften (HAW) Hamburg, 22087 Hamburg, Germany; markus.oberthuer@haw-hamburg.de

**Keywords:** aminolysis, textiles, PET, functional coatings, polymer brushes

## Abstract

Here, we synthesize zwitterionic polymer brushes on polyester fabrics by atom transfer radical polymerization (ATRP) after a prefunctionalization step involving an aminolysis reaction with ethylenediamine. Aminolysis is an easy method to achieve homogeneous distributions of functional groups on polyester fibers (PET) fabrics. Varying the polymerization time and the prefunctionalization conditions of the reaction, it is possible to tune the amount of water retained over the surface and study its effect on protein adhesion. This study revealed that the polymerization time plays a major role in preventing protein adhesion on the PET surface.

## 1. Introduction

Biofouling is a problem affecting the efficacy of a broad range of systems and applications. These include implants, biosensors, or textiles used in the medical sector, where the contamination by bacteria and microorganisms is a major cause of infections [1,2,3,4].

Although in the food industry and in the healthcare sector this problem is addressed by using strict hygiene standards, for several other applications, a limitation of the interactions with bacteria and fouling is not always achievable. In addition, the increased resistance of bacteria to antibiotics and biocides has led many researchers to drive their attention to antifouling surfaces rather than antibacterial surfaces [3,4,5,6].

In recent years, lubricant-infused surfaces have been found to reduce the lateral adhesion of all types of liquids and to limit bacteria and microorganisms surface interactions, although such surfaces often involve the use of non-environmentally friendly compounds [7,8,9,10,11].

A major drawback concerning these surfaces is that liquid lubricants are easily removed by shear stress or evaporation. Several strategies have been adopted to limit the depletion of the lubricant, yet further efforts are needed to improve the performances of slippery surfaces. A long-term stability might be achieved using highly hydrated polymer brushes to replace the lubricating layer. Hydration is a fundamental aspect when choosing a polymer that can prevent the attachment of proteins or bacteria. The interactions of the polymeric chain with water molecules in fact inhibits the non-polar interactions between a surface and a bacteria cell envelope [12]. In addition, steric hindrance plays an important role in avoiding protein adhesion, which is a reason why not all hydrophilic surfaces are able to prevent fouling [13,14,15]. Functionalization by polymers implies either a surface grafting of the polymer on a surface (grafting to) or a polymerization using a “grafting from” approach. The latter is often preferred when a better control over grafting density is needed [16,17,18,19,20,21,22,23].

Among all polymers, polyethylene glycol (PEGs) and zwitterionic polymers proved to be highly effective against the adhesion of foulants, since several coatings that prevent the attachment of proteins and bacteria have already been synthesized [24,25]. PEG and PEG derivatives were widely used in many applications, including implantable surfaces or the functionalization of nanoparticles (NPs) for drug delivery, where these systems could prolong the blood circulation time [26,27,28,29,30,31,32,33,34,35,36]. However, oxidative damage of PEGylated surfaces and PEG’s non-biodegradability represents a major concern and limits its use to short-term applications [37].

On the other hand, zwitterionic polymers proved to be very promising for such applications. Zwitterions are neutral but charged molecules containing both a positively and a negatively charged site [38,39]. This charge separation results in a pronounced hydration effect due to the strong interactions with water molecules [25,40].

Among zwitterionic polymers, sulfobetaine methacrylate (SBMA) polymers showed low fouling properties and have attracted considerable attention also due to an upper critical solution temperature (UCST) behavior [41,42,43,44,45,46,47].

The importance of realizing such antifouling surfaces extends to textile materials as well. For example, textiles used in filtration systems are extensively compromised by fouling, which lowers their durability and requires them to be replaced at short-term distances. Among all fibers, polyester fibers (PET) are broadly used because of their good workability and low price. In addition, it is possible to control filtrations properties by the fiber diameter, the mesh size, and the absolute material thickness [48]. The functionalization of polyester is very demanding due to its lack of functional groups within the polymer structure. Several strategies might be adopted in order to increase the reactivity of PET substrates adding functional groups. These include the alkaline hydrolysis of PET sheets, the plasma activation of textile surfaces, or the immobilization of adhesive polymers rich in functional groups [42,48,49,50]. Long-term stability and the homogeneous distribution of these groups are crucially important to ensure a stable and efficient functionalization of the substrates. Timma et al. recently reported in their work how to tune lipase adhesion on PET and cotton, varying the degree of substitution of zwitterionic sulfobetaines on polyvinylamine as the scaffold. In this scenario, the density of the grafted polyzwitterions is determined by the concentration of the polyzwitterions during the reaction process [51]. However, it must be considered that this approach might fail in achieving a homogeneous distribution of the polymer chains onto the fibers, since the adsorption of polyvinylamine on PET is influenced by a number of factors [52]. When synthesizing polymer brushes, the surface distribution of the functional groups available for the functionalization affects the result dramatically. Therefore, in our work, we use an alternative method to achieve an even and homogeneous distribution of functional groups available for a subsequent functionalization. Aminolysis using several amines is a reaction involved in the recycling of polyester fibers, yet, if conducted in controlled conditions, it is possible to homogeneously anchor functional groups to the PET surface [53,54,55,56,57,58,59,60].

The advantage of using aminolysis as a prefunctionalization step lies in the possibility to tune the density of the functional groups on the surface in order to control the grafting density of the immobilized polymers in the postfunctionalization step. This was achieved by varying the reaction time of aminolysis. Consequently, the polyester fabrics were functionalized with polysulfobetaine brushes testing different polymerization times, and their anti-adhesive properties were characterized. The high motility of the polymer chains and the strong hydration effect associated with the charges make these materials among the best candidates for the fabrication of environmentally friendly antifouling textile surfaces.

## 2. Materials and Methods

### 2.1. Materials

Woven PET fabrics, area weight of 170 (g·m^−2^), were purchased by wfk Testgewebe GmbH. The reagents ethylenediamine (EDA, 99.5%), 2-(dimethylamino)ethyl methacrylate (DMAEMA, 98%), 1,3-propanesultone, [2-(methacryloyloxy)ethyl]dimethyl-(3-sulfopropyl) ammonium hydroxide (SBMA, 95%), 2-bromoisobutyryl bromide (BIBB, 98%), Triethylamine (TEA, 99%), 2,2’-bipyridyl (≥ 99%), Copper(I) bromide (CuBr, 99.9%), and copper (II) bromide (CuBr_2_, 99.9%) were purchased from Sigma-Aldrich (Steinheim, Germany). Ethanol 99% denatured with 1% MEK technical grade was purchased by AppliChem GmbH (Darmstadt, Germany). Tetrahydrofuran (THF) and methanol were purchased by Carl Roth GmbH (Karlsruhe, Germany). All PET textiles were extracted using a Soxhlet apparatus with ethanol–water (1:1 *v/v*, 8 h) and petroleum ether (8 h) to remove production-related finishes.

### 2.2. Prefunctionalization of PET Fabrics by Aminolysis Reaction

PET fabrics were cut into round pieces of 3-cm diameter and put in a flask filled with a 40% *v/v* solution of ethylenediamine in ethanol. The flask was heated at 55 °C for a time range of 15 min to 3.5 h in order to achieve different functional groups densities. Then, the samples were washed with ethanol and water to remove unbound ethylenediamine. Then, the PET_EDA samples were obtained.

### 2.3. Immobilization of Initiator

PET_EDA samples were immersed in a flask containing 50 mL of THF. Then, 1.1 mL (9.5 mmol) of BIBB as initiator and 0.7 mL (5 mmol) of TEA were added to the flask.

The reaction occurred under nitrogen atmosphere for 24 h. The samples PET_Br were extensively washed with THF and dried at room temperature to remove by-products.

### 2.4. ATRP Polymerization

A nitrogen-purged Schlenk flask was loaded with 5 mL of a 4:1 *v/v* methanol-water, 0.312 g (2 mmol) of bipyridyl, 0.144 g (1 mmol) of CuBr, and 0.0111 g (0.05 mmol) of CuBr_2_.

Then, 10 g (35 mmol) of SBMA were added to the mixture and the solution was stirred under nitrogen.

PET_Br samples were put in separate flasks and air was removed by purging nitrogen through a syringe. The polymerization mixture was added and left at room temperature. The polymerization time varied from 1 to 24 h.

After polymerization, PET_PSBMA samples were washed with ethanol and dried overnight.

### 2.5. Characterization

ATR-FTIR measurements were performed on a coupled with an ATR accessory (Shimadzu, Kyoto, Japan). An IR Prestige (Shimadzu, Kyoto, Japan) equipped with a golden gate, diamond crystal (Specac, Orpington, UK) with a resolution of 4 cm^−1^ was used. The spectra were recorded over 100 scans. The quantitative determination of functional groups onto PET fabrics was conducted using a Bruker Vertex 70 FTIR spectrometer (Bruker Optic GmbH, Ettlingen, Germany). The morphology of the brushes-modified PET fabrics was observed using scanning electron microscopy (SEM, S-3400 N II, Hitachi High Technologies Europe GmbH, Tokyo, Japan). Before the measurement, the surfaces of the modified PET fabrics were sputtered with gold in vacuum for 4 min with a sputter coater (Quorum Emitech K500X, Ashford, Kent, UK). The surface topography and phase of the modified fabrics was also characterized using scanning probe microscopy (SPM, Agilent Technologies, Santa Clara, CA, USA). The measurements were conducted with an Agilent Technologies 5500 SPM using a Nanosensor tapping-mode cantilevers with silicon tips (AC frequency 146–236 kHz, length 225 ± 10 μm, width 38 ± 7.5 μm, and thickness of 7 ± 1 μm, force constant 21–98 N/m, tip height 10–15 μm). The resolution of all images was set at 1024 data points with a scanning speed of 0.5 lines per second. All captured images (5 µm × 5 µm) were processed by Gwyddion software (Open-source software developed by Department of Nanometrology, Czech Metrology Institute, Brno, Czech Republic). After data leveling, the polynomial background was subtracted to remove the curvature using the second-order polynomial.

Then, the roughness parameter *R*q (RMS) was obtained by the average of the row/column roughness statistics of the image at maximum resolution.

The roughness parameters *R*q (root mean square (RMS)) were obtained of the whole image at maximum resolution.

### 2.6. Determination of Functional Groups

Functional group densities were calculated by colorimetric methods using Orange II dye according to an established method [54]. In brief, aminolyzed PET samples were immersed in aqueous solutions of Orange II and adjusted to pH = 3. Acidification is crucial, since amino and hydroxyl groups must be protonated in order to retain the anionic dye. Then, the solutions were heated to 50 °C for 20 min, and the samples were subsequently rinsed using an acidic solution to remove the unbound dye.

Once dried, the colored PET_EDA samples were immersed in a basic solution in order to release the dye. These solutions were adjusted at pH = 3, and their absorbance was measured at 484 nm to estimate the concentration of the functional groups.

### 2.7. Determination of Protein Adsorption

Lipase was chosen as a reference protein, since it is able to cleave fluorescein diacetate into fluorescein. Therefore, it is possible to measure the fluorescence intensity of the system in order to calculate the amount of protein absorbed on PET.

The procedure to assess protein adhesion on PET fabrics is described elsewhere [61]. In brief, the fabrics (d = 15 mm) were incubated in a lipase solution (type VII from Candida rugosa, 1 g·L^−1^ in 100 mM phosphate buffer pH = 7.2) and placed on a shaker at 50 rpm for 30 min.

The unbound lipase was washed, placing the samples in deionized water for 1 min. Then, the samples were dried and transferred into a 24-well plate. Then, 500 mL of phosphate buffer were added to each well. The plate was placed in a TECAN plate reader with an injection unit. Then, 50 µL FDA (2 mg·mL^−1^ in DMF) were added to each well automatically; after 15 s of shaking, the fluorescence intensity (absorption 485 nm, emission 535 nm) was measured.

## 3. Results and Discussion

### 3.1. PET Prefunctionalization by Aminolysis

**Figure 1:**
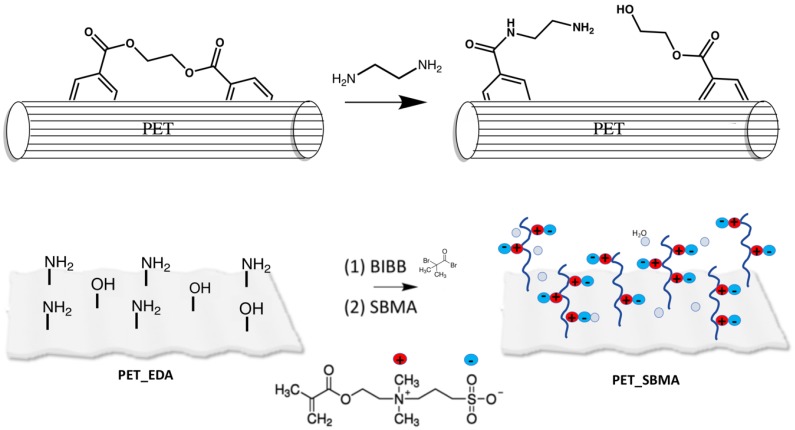
Scheme of synthesis of poly(sulfobetaine methacrylate) (PSBMA) brushes on a polyester fibers (PET) fabric.

The aminolysis reaction performed on PET is illustrated in Figure 1. By aminolysis, amines are covalently bonded to the PET surface, implying a breakup of the polyester bonds and the formation of hydroxyl groups. In our study, a concentration of ethylenediamine (EDA) and ethanol of 40:60 *v/v* and a temperature of 55 °C were used as set conditions for every prefunctionalization step, and time was varied in order to study the grafting process of amino groups on PET.

**Figure 2:**
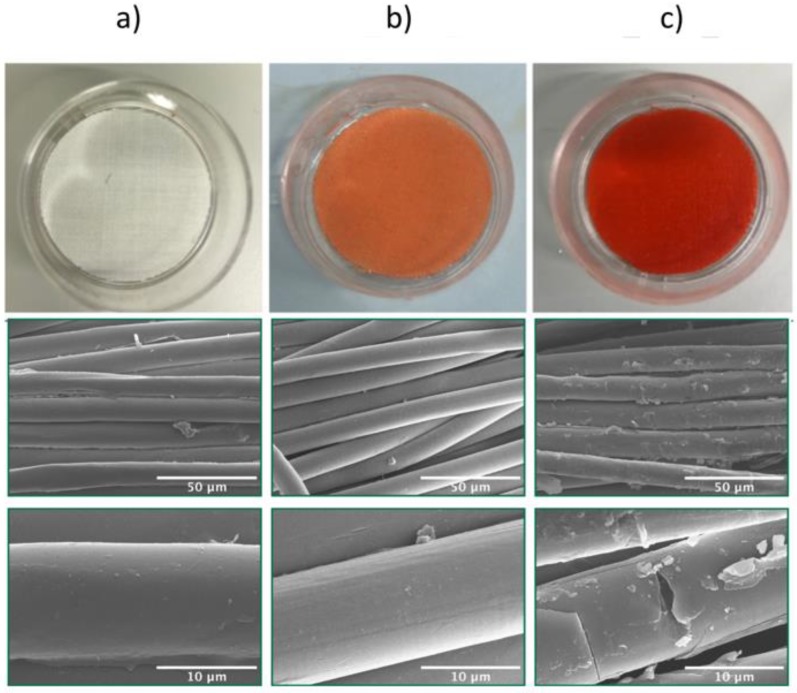
Samples reacted with Orange II and SEM images of (**a**) Pristine PET, (**b**) PET aminolyzed with ethylenediamine (EDA) for 1 h, (**c**) PET aminolysed with EDA for 3 h.

By this approach, it is possible to tune the density of the functional groups on the surface of PET by varying the reaction time. When the reaction is conducted in critical conditions, the polyester fibers are completely dissolved in the medium, as the reaction implies the fragmentation of the polyester chain [61]. Thus, a kinetic study of the aminolysis reaction is essential to control the grafting of functional groups and retain the mechanical performances of the material itself. As an example, we report here a preliminary study of the aminolysis reaction on polyester fabrics. Figure 2 shows pristine PET (a), PET aminolyzed for 1 h (b), and PET aminolyzed for 3 h (c), respectively. After the reaction, the samples were dyed using a solution of Orange II. Orange II is an anionic dye that is capable of binding amino groups in acid conditions and therefore can be an useful tool to assess the degree of functionalization of the polyester fabric [54]. In Figure 2, SEM images show the appearance of a native PET fiber and the PET fibers of samples aminolyzed respectively for 1 h and 3 h. The surface of the pristine PET fiber was initially smooth. After 1 h, the fibers kept retaining their smoothness, although a considerable degree of surface functionalization was achieved. As long as the reaction proceeded, amino groups were introduced on the PET surface. As studied in previous works, the concentration of the ethylenediamine solution, reaction time, as well as temperature of the reaction are all influencing the mechanical properties of the native PET [61,62].

**Figure 3:**
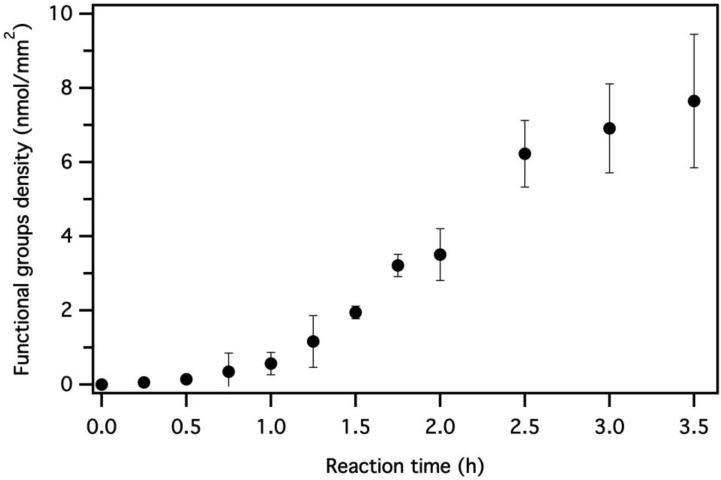
Amino groups density increases over time. The geometrical surface of the PET samples was used to calculate the surface groups density.

Figure 3 shows the results of the quantitative analysis of the amino groups density over time, which was conducted by using Orange II dye according to the procedure reported above [54]. Please note that the geometrical surface of the PET samples was used to calculate the surface amino groups density. An increase in the reaction time results in highly functionalized PET fabrics. The saturation time of the process corresponds to 3.5 h, after which the samples were completely dissolved in the medium.

### 3.2. Synthesis of SBMA Polymer Brushes on PET

**Figure 4:**
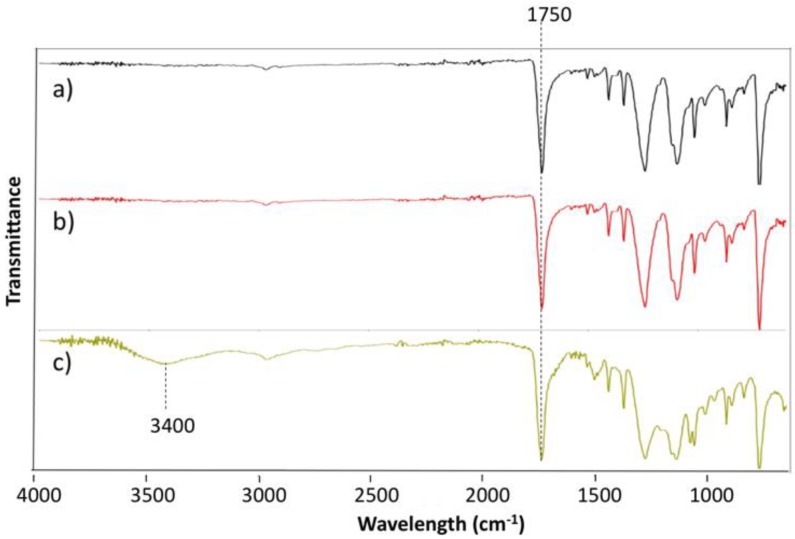
ATR-FTIR spectra for (**a**) PET, (**b**) PET aminolyzed with EDA (PET_EDA), and (**c**) PET functionalized with poly(sulfobetaine methacrylate) (PET_PSBMA).

Atom transfer radical polymerization (ATRP) was used to synthesize polymer brushes on PET fabrics. It must be noted that the immobilization of the initiator onto the surface was conducted with an excess of BIBB in order to saturate all the amino and hydroxyl groups formed on the surface. Figure 4 shows the ATR-FTIR spectra of pristine PET, PET aminolyzed (PET_EDA) for 1 h, and PET functionalized with sulfobetaine methacrylate polymer brushes (PET_PSBMA) for 24 h. All spectra reveal the appearance of a peak at 1750 cm^−1^ corresponding to the stretching vibration of the ester carbonyl group. The successful synthesis of poly(sulfobetaine methacrylate) (PSBMA) was confirmed by the appearance of the O–H stretching band at 3400 cm^−1^. Although PSBMA does not have any hydroxyl group in the molecular structure, the presence of an O–H band indicates an interaction of the charged sites of PSBMA with water.

**Figure 5:**
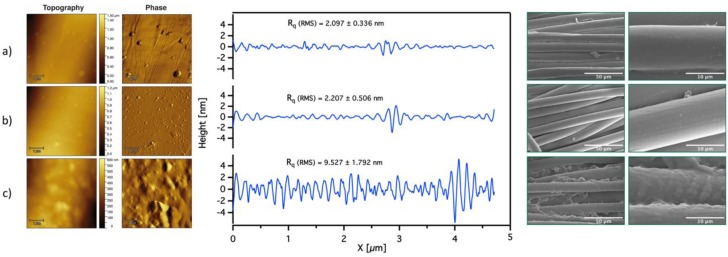
(**a**) AFM images with roughness profiles and SEM images of (**a**) PET; (**b**) PET functionalized with EDA (PET_EDA); and (**c**) PET functionalized with PSBMA (PET_PSBMA).

Pristine PET and functionalized PET were also analyzed by SEM and AFM, as shown in Figure 5. The appearance of the fibers did not change after the aminolysis reaction with EDA. After functionalization of the PET with SBMA polymer brushes, the fibers were no longer smooth and were covered by a dense coating. AFM topography and phase images were acquired in tapping mode with a scanning area of 5 µm × 5 µm and shown in Figure 5. These confirmed an increase in the roughness of the PET_PSBMA fibers compared to those of pristine PET. *R*q (RMS) values for PET, PET_EDA, and PET_PSBMA were found to be 2.097 ± 0.336 nm, 2.207 ± 0.506 nm, and 9.527 ± 1.792 nm respectively. Therefore, no significant change is observed in the roughness profile of PET and PET after aminolysis, yet the roughness increases after functionalization with PSBMA.

**Figure 6:**
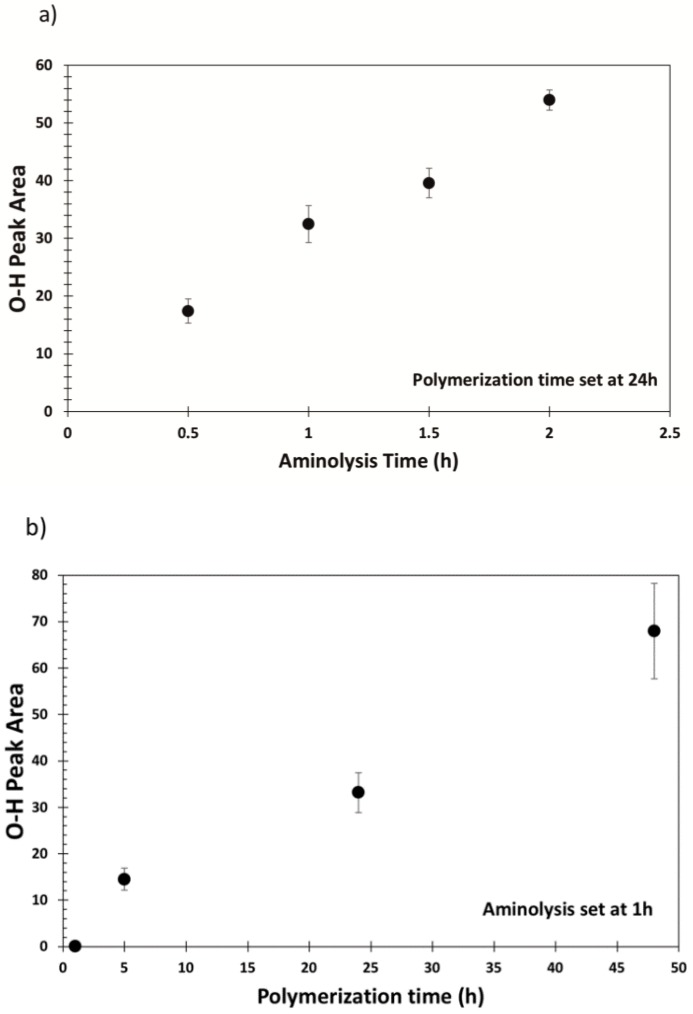
Water O–H stretching band area of samples obtained at (**a**) different aminolysis reaction times and (**b**) different polymerization times.

Figure 6 reports the area of O–H stretching bands in an ATR-FTIR spectrum resulting from the interaction of the polymer brushes with water present in the air. Data belong to samples aminolyzed for respectively 0.5, 1, 1.5, and 2 h and functionalized with PSBMA brushes for 24 h (Figure 6a) and samples aminolyzed for 1 h and functionalized by setting the polymerization time respectively to 1, 5, 24 and 48 h (Figure 6b). For the former, 24 h was chosen as the reference polymerization time, while for the latter, the aminolysis reaction time was set at 1 h since at this stage the fibers are intact, yet show a significant level of functionalization (as shown in Figure 2 and Figure 3).

A variation of the aminolysis reaction time alters the degree of functionalization of PET fibers and thus changes the density of the polymer chains, while the polymerization time controls the length of the polymer chains. As a consequence, longer polymer chains can allocate more water molecules, allowing a formation of a lubricating layer. This is supported by an increase in the area of the absorbed water O–H stretching band in both cases.

This result confirms that the amount of water interacting with the polymer chains can be effectively tuned by changing either the aminolysis time or the polymerization time.

**Figure 7:**
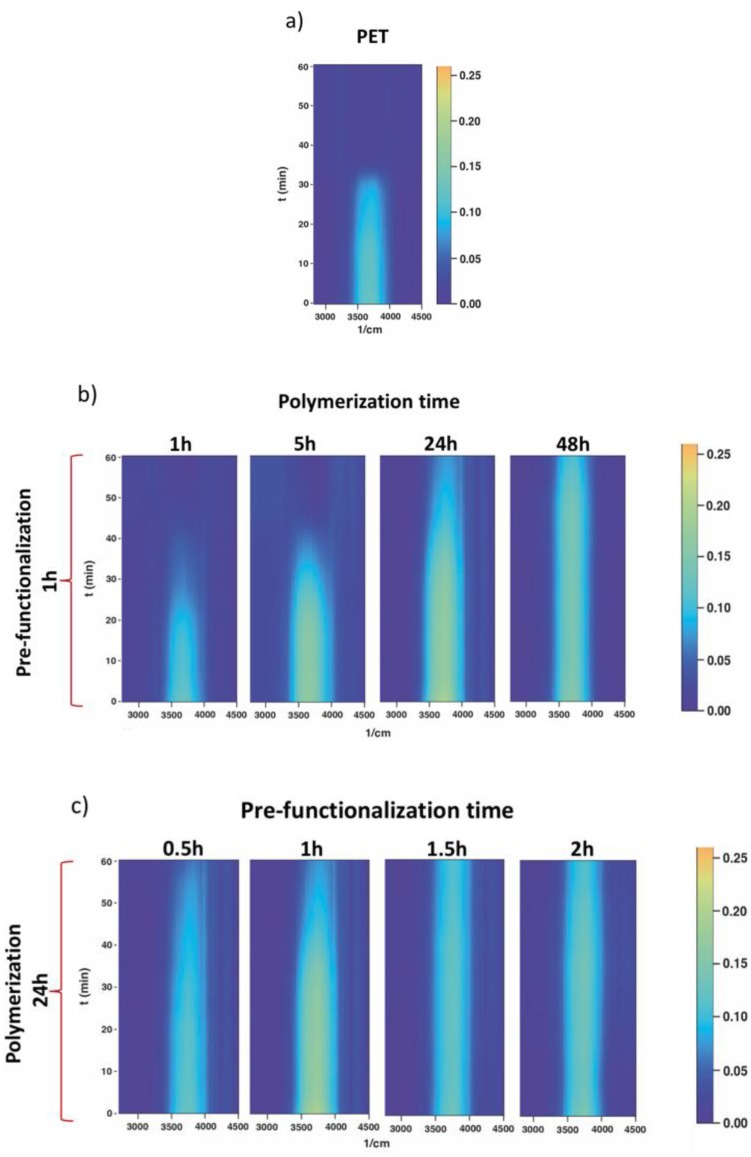
Contour plots obtained by the interpolation of time dependent ATR-FTIR analyses on wet samples.

Figure 7 shows water retention contour plots of pristine PET (a) compared to samples obtained by varying the polymerization time (b) and aminolysis reaction time (c). The plots were processed by the interpolation of time-dependent ATR-FTIR spectra acquired on wet samples in a range of 60 min with a focus on the frequency range of 2500–4500 cm^−1^. In this region, the light blue band between 3400 cm^−1^ and 4000 cm^−1^ corresponds to the water O–H stretching band in an FTIR spectrum. Samples were wetted before performing the analyses in order to monitor the variation of the O–H band intensity over time, and then to get an insight into the capability of the different PET_PSBMA samples to retain water.

In Figure 7c, samples aminolyzed for 1.5 h and 2 h still show intense water retention after 60 min, while for those aminolyzed for 0.5 h and 1 h, the O–H band slightly start to fade after 40 min.

In Figure 7b, the water retention time increases as polymerization time increases. After 1 h of polymerization, no increase is observed in comparison with pristine PET, while a significant change was observed, setting the polymerization time to 5 h. This can be explained by considering the correlation between the polymerization time and chain length. Higher polymerization times allow increasing the molecular weight of the polymer brushes. The water bound on the charged sites of the polymeric chains takes more time to evaporate if the molecular weight of the chains is higher.

Overall, the polymerization time has a greater effect than the aminolysis reaction time.

These results show that in the investigated brushes’ density range, a variation of the polymerization time results in a variation of the water retention on the PET fabric. Therefore, polymerization from low dense surfaces, i.e., obtained from low aminolysis reaction times, are still sufficient to retain a significant amount of water compared to the native fabric.

### 3.3. Protein Adhesion

**Figure 8:**
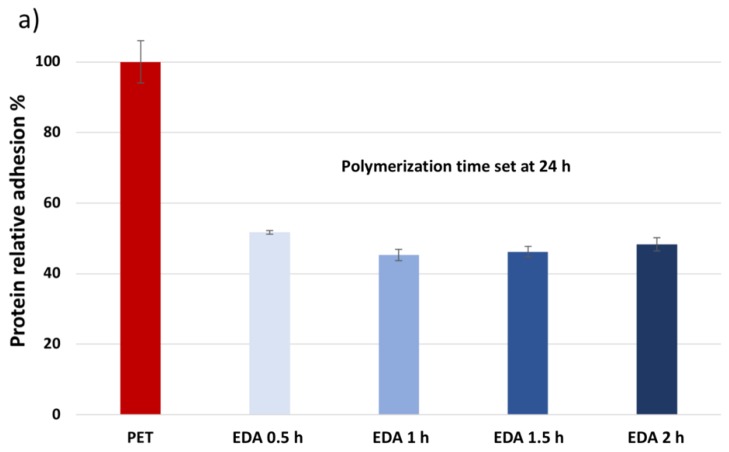

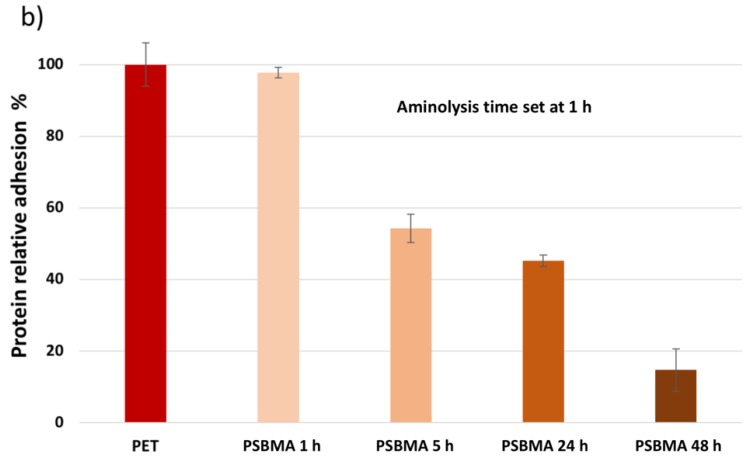
Results of protein adhesion tests of native PET and PET (**a**) aminolyzed at different times and polymerized for 24 h, and (**b**) aminolyzed for 1 h and polymerized at different times.

Protein adhesion tests were performed on the native PET and on the functionalized PET samples. Figure 8 reports the results of lipase adhesion on samples obtained at different aminolysis times and different polymerization times. Figure 8a shows that a change in the aminolysis reaction time does not influence the adsorption of lipase on the surface of the textiles, as there is no significant difference in the adsorption on samples aminolyzed at different times. It must be pointed out that since protein adhesion tests were performed in water, it is expected for the polymer brushes to adopt a stretched conformation [63]. Although density plays a role in the thickness of grafted polymers, in this scenario, the examined density range does not considerably affect the results in the adhesion measurements. In Figure 8b, the adhesion was measured on samples obtained by changing the polymerization time. Samples where both aminolysis and the polymerization time was set at 1 h show no reduction in protein adhesion. A decrease in adhesion to nearly 80% is registered as the polymerization time increases. Therefore, the polymerization time seems to play a major role in the proteins’ adsorption.

## 4. Conclusions

We have synthesized zwitterionic polymer brushes on PET fabrics by means of a chemical prefunctionalization involving aminolysis followed by ATRP. A variation of the aminolysis reaction time and the polymerization time led to surfaces with distinct water retention behaviors that resulted from respectively a difference in the distribution and length of the polymer brushes on the PET surface. Accordingly, aminolysis is a fast and simple way to tune the density of a wide selection of polymers that can be grafted onto PET.

Adhesion tests were performed on native PET and PET functionalized with PSBMA brushes having different densities and grown at different polymerization times. In this scenario, the polymerization time has a higher impact on the antiadhesive properties of PET compared to density. Since the polymerization time is proportional to the molecular weight of the polymer brushes, a high polymerization time led PET_PSBMA to allocate more water molecules, therefore limiting the interaction with lipase.

Understanding how to optimize the reaction conditions of the functionalization processes on textiles is a key element in order to upscale the production of antifouling fabrics that may be used in several industrial applications, including filtration systems, membranes, or in the biomedical sector.
